# Case Report: Partial Uniparental Disomy Unmasks a Novel Recessive Mutation in the *LYST* Gene in a Patient With a Severe Phenotype of Chédiak-Higashi Syndrome

**DOI:** 10.3389/fimmu.2021.625591

**Published:** 2021-03-31

**Authors:** Mireia Boluda-Navarro, Mariam Ibáñez, Alessandro Liquori, Clara Franco-Jarava, Mónica Martínez-Gallo, Héctor Rodríguez-Vega, Jaijo Teresa, Carmen Carreras, Esperanza Such, Ángel Zúñiga, Roger Colobran, José Vicente Cervera

**Affiliations:** ^1^ Accredited Research Group in Hematology and Hemotherapy, Instituto de Investigación Sanitaria La Fe, Valencia, Spain; ^2^ Department of Hematology, Hospital Universitario y Politécnico La Fe, Barcelona, Spain; ^3^ Centro de Investigación Biomédica en Red de Cáncer (CIBERONC), Madrid, Spain; ^4^ Department of Medicine, University of Valencia, Valencia, Spain; ^5^ Departamento de Ciencias Biomédicas, Facultad de Ciencias de la Salud, Universidad CEU Cardenal Herrera, Valencia, Spain; ^6^ Immunology Division, Hospital Universitari Vall d'Hebron (HUVH), Diagnostic Immunology, Vall d’Hebron Research Institute (VHIR), Barcelona, Spain; ^7^ Department of Cell Biology, Physiology and Immunology, Autonomous University of Barcelona (UAB), Barcelona, Spain; ^8^ Pediatric Hematology Unit, Hospital Universitario y Politécnico La Fe, Valencia, Spain; ^9^ Genetics Unit, Hospital Universitario y Politécnico La Fe, Valencia, Spain; ^10^ Department of Clinical and Molecular Genetics, Hospital Universitari Vall d’Hebron (HUVH), Barcelona, Spain

**Keywords:** Chédiak-Higashi syndrome, CHS, primary immunodeficiency, hemophagocytic lymphohistiocytosis, *LYST*, SNP-array, uniparental disomy, loss of heterozygosity

## Abstract

Chédiak-Higashi syndrome (CHS) is a rare autosomal recessive (AR) immune disorder that has usually been associated to missense, nonsense or indels mutations in the *LYST* gene. In this study, we describe for the first time the case of a CHS patient carrying a homozygous mutation in the *LYST* gene inherited as a result of a partial uniparental isodisomy (UPiD) of maternal origin. Sanger sequencing of the *LYST* cDNA and single nucleotide polymorphism (SNP)-arrays were performed to identify the causative mutation and to explain the molecular mechanism of inheritance, respectively. Partial-UPiD leads to a copy neutral loss of heterozygosity (CN-LOH) of the telomeric region of chromosome 1 (1q41q44), unmasking the potential effect of the mutation detected. The mutation (c.8380dupT) is an insertion located in exon 32 of the *LYST* gene resulting in a premature stop codon and leading to the loss of all the conserved domains at the C-terminal of the LYST protein. This would account for the severe phenotype observed. We also reviewed the only two previously reported cases of CHS as a result of a uniparental disomy. In this study, we show that the combination of different strategies, including the use of SNP-arrays, is pivotal to fine-tune the diagnosis of rare AR disorders, such as CHS. Moreover, this case highlights the relevance of uniparental disomy as a potential mechanism of CHS expression in non-consanguineous families.

## Introduction

Uniparental disomy (UPD) is an unusual genetic mechanism in which homologous copies of a specific chromosome are inherited from only one parent (1). This event can occur as hetero- (UPhD) or iso-disomy (UPiD) depending on whether the two chromosomes transmitted are different or identical, respectively (2). Furthermore, UPiD can be partial (partial-UPiD) when only a fragment of a chromosome is affected.

UPD does not always turn out in disease, thus, the clinical outcome is directly related to the genetic content, the size of the affected chromosomal region, and the degree of mosaicism (3, 4). On this regard, abnormal phenotypes associated to UPD can result from the presence of imprinted genes on the chromosome involved by altering their regulation and dosage due to variations in epigenetic marks (5). UPD can also be implicated in autosomal recessive (AR) diseases by uncovering a recessive disease allele (6–9).

CHS (OMIM #214500) is a rare AR immune disorder characterized by immunodeficiency and severe hemophagocytic lymphohistiocytosis caused by altered functional cytotoxic lymphocytes, protein missorting in neutrophil and loss of natural killer (NK) cell function. CHS patients typically present oculocutaneous albinism, thin silvery white skin, a predisposition to bleeding, neurological dysfunction and infections, among others. In a morphological analysis, CHS is detected by the presence of large pathognomonic cytoplasmic lysosomal vesicles in granulocytic cells resulting from the dysregulated function of lysosomes (10). CHS immune dysfunction is linked to infections and/or lymphoproliferative disorders with a poor prognosis unless treated by allogeneic bone marrow transplantation (11).

CHS is caused by mutations in the lysosome trafficking regulator (*LYST)* gene, also known as *CHS1*, which is localized on chromosome 1q42.1-q42.2 (12). The *LYST* gene encompasses 53 exons and encodes for the LYST protein, which contains several highly conserved (BEACH, PH and WD40) domains at the C-terminal end. Even though the exact function of the LYST protein is still debated, the combined PH-BEACH motifs are thought to be implicated in different aspects of vesicular trafficking and, thus, likely playing a crucial role in regulating lysosome-related organelle size, fission and secretion (12–14). Both homozygous and compound heterozygous mutations in *LYST* gene have been previously described in CHS (10). Loss-of-function mutations are associated with the severe CHS childhood form leading to death if untreated. In contrast, missense mutations are observed in patients with a milder form of the disease, allowing survival into adulthood (11).

Here, we report the first case of CHS resulted from a partial-UPiD in a young patient harboring a novel mutation in the *LYST* gene. The mutation was present in homozygosis despite segregation analysis revealed the mother as the only carrier. Subsequent single nucleotide polymorphism (SNP)-arrays analysis allowed us to identify a segmental isodisomy of maternal origin as the cause of this uncommon genetic inheritance.

## Case Presentation

Here we present a 3-year-old girl, born from non-consanguineous parents after a normal pregnancy and cesarean delivery, hospitalized after 2 days of high fever, productive cough, clear nasal secretions and lack of appetite. One week before, she had suffered a non-febrile respiratory infection treated with azithromycin. She presented incomplete ocular albinism characterized by absence of pigmentation in the peripheral retina but had no history of serious recurrent infections or excessive bleeding. There was no family history of albinism or hypopigmentation, immunological or hematological alterations, or of deaths at an early age.

### Clinical, Laboratory and Genetic Results

**Figure 1:**
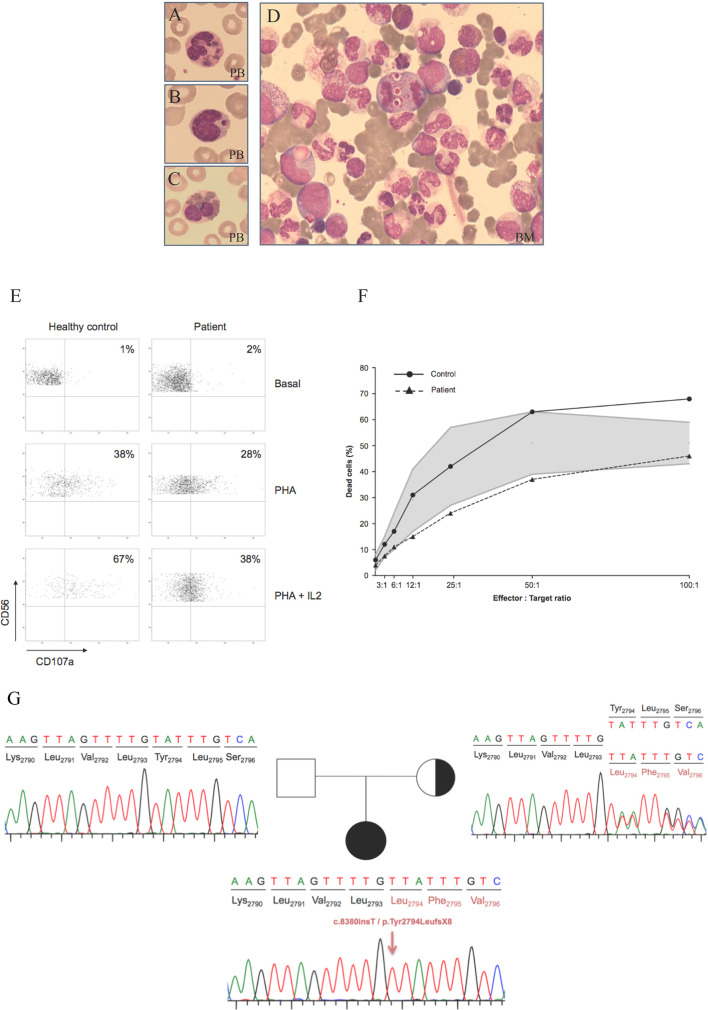
Morphological, cellular and molecular findings in the CHS patient. **(A–C)** Wright stains of peripheral smear showing a neutrophil, lymphocyte and eosinophil with giant intracytoplasmic granules. **(D)** Bone marrow aspirate. Intense vacuolization and purple inclusions are also observed in immature cells of the myeloid linage. PB: peripheral blood; BM: bone marrow. **(E)** Degranulation assay was decreased compared to the healthy control both with PHA and PHA + IL2. PBMCs were incubated with or without (resting cells) PHA either alone or with IL-2 for 4 hr at 37°C. Thereafter, cells were stained with fluorochrome-conjugated anti-CD56, and anti-CD107a mAbs. CD107a surface expression was gated on CD56 cells. **(F)** A flow cytometry-based NK cytotoxicity assay was performed by measuring cytotoxicity against K562 cell line in a 4-hour staining with propidium iodide. The shaded area indicates normal ranges of cytotoxicity tested in healthy individuals. The patient showed a cytotoxic function on the lower range of normality. **(G)** Family tree and sequence analysis of the patient and his parents. Sequence patterns showing the patient’s mutation c.8380dupT in homozygosis, the normal sequence of the non-carrier father and the mother’s sequence showing the mutation in heterozygosis.

During her evaluation, pancytopenia and oculocutaneous albinism were identified whilst chest x-rays (CXR) did not show significant alterations. Infectious diseases were ruled out by serological screening and direct observation in blood smears. Analysis for respiratory viruses was positive for adenovirus and respiratory syncytial virus (RSV). She showed normal psychomotor development and her vaccination schedule was up to date. Morphological studies were performed both on peripheral blood (PB) and bone marrow (BM) samples. Results for PB cells were consistent with CHS alterations. Hypercellular BM with increased levels of the granulocytic series and lower erythroid and lymphocytes series was also observed. In PB as in BM, purple inclusions were detected in both lymphocytes and all stages of the granulocytic series. Intense vacuolization, sometimes with a purple inclusion, was also seen in all cell types (**Figures 1A–D**). BM’s flow cytometry showed a normal immunophenotype, with an increase of immature myeloid cells (**Supplementary Table 1**). NK cells showed defective granule exocytosis with low expression of CD107a on stimulated NK cells (**Figure 1E**) and a slightly reduced cytotoxic capacity (**Figure 1F**) evaluated by degranulation assay.

**Figure 2:**
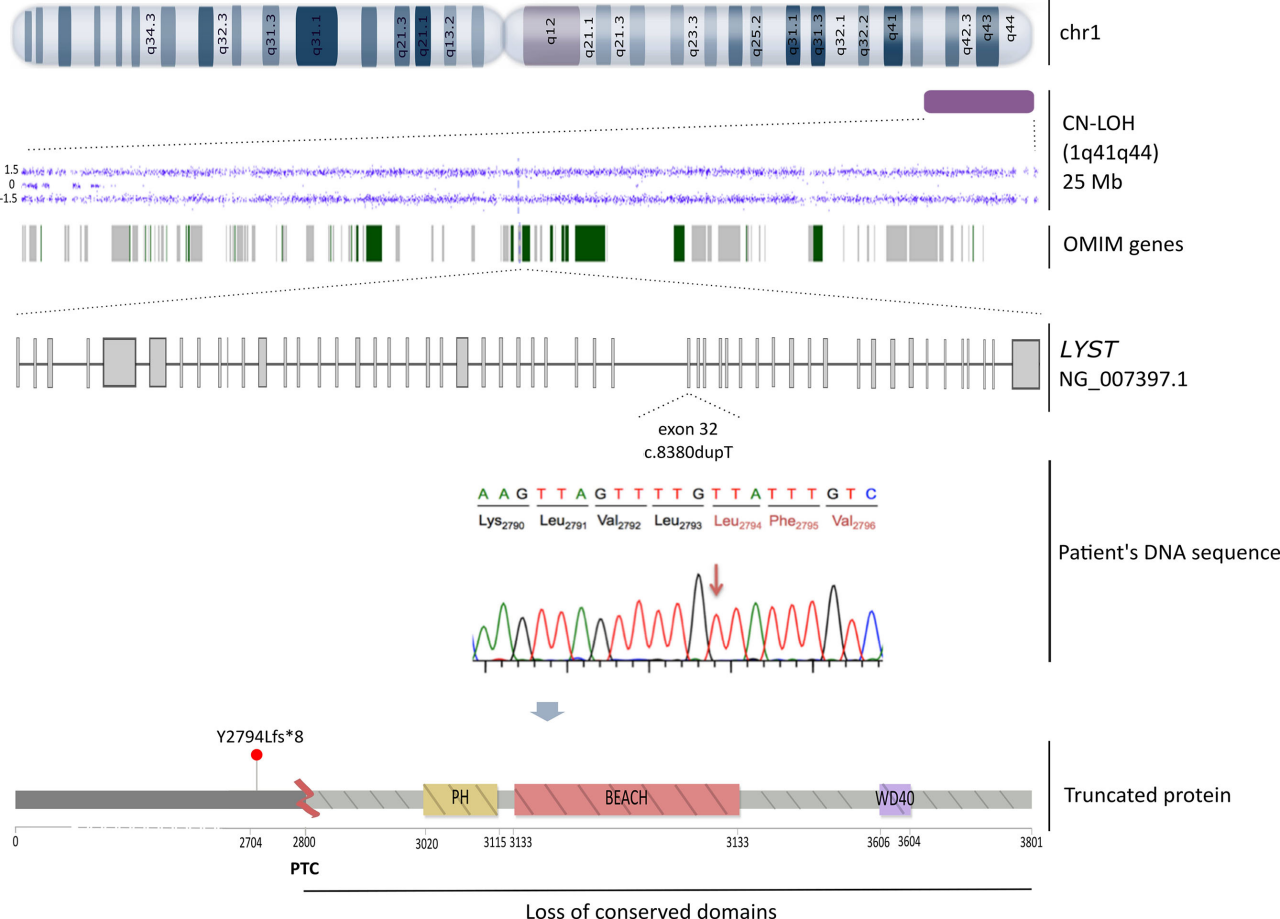
Schematic representation of the triggering molecular mechanism of CHS in our patient. From top to bottom: the CN-LOH detected by SNP-array (Cytoscan HD, Affymetrix) in chromosome 1 (1q41q44) is delineated as a purple rectangle, the dots depict the altered markers in the array and the green and grey bands represent the 93 OMIM genes affected, including the *LYST* gene. Below, the *LYST* gene (NG_007397.1) that encodes for the entire functional protein is depicted. The c.8380dupT (NM_000081.3) frameshift mutation is localized in the exon 32. A lollipop-like (15) image reflects the resulting truncated protein owing to a PTC at 2,800 amino acid that would remove all conserved domains (PH, BEACH and WD40).

Differential diagnosis by cytogenetic analysis, including conventional karyotype by C-banding and FISH revealed a normal female karyotype (46,XX). Due to the clinical phenotype that included albinism, together with the defective degranulation assay and the presence of cytoplasmic lysosomal vesicles in granulocytic cells, we amplified by PCR the entire patient’s cDNA sequence of *LYST* gene (53 exons) in 5 overlapping fragments (**Supplementary Table 2, Supplementary Figure 1A**). PCR products were then sequenced by Sanger. The genotype analysis of the patient revealed a homozygous frameshift c.8380dupT (NM_000081.3) variant in the exon 32 (**Figure 1G**) that would lead to a premature stop codon (PTC) (NP_000072.2: p.Tyr2794Leufs*8). The presence of this variant was then confirmed by the targeted sequencing of the corresponding genomic region (gDNA). The *LYST* c.8380dupT variant was not reported in the literature or in the main population databases (e.g. dbSNP, ExAC and gnomAD). The effect of the variant on the encoded protein was assessed by identifying and comparing conserved domains in both the reference and the resulting mutated LYST protein with the NCBI Conserved Domain Search Tool (https://www.ncbi.nlm.nih.gov/Structure/cdd/wrpsb.cgi) (**Figure 2**). This tool revealed that the variant identified might remove all conserved domains responsible for the protein function (PH, BEACH and WD40). Given the analysis performed on the variant effect and the patient phenotype, we have classified this variant as pathogenic (PVS1 according to the American College of Medical Genetics and Genomics recommendation) (16). Written informed consent from the parents was provided for all genetic studies reported here.

To determine the inheritance pattern, segregation analysis was performed revealing only the mother as the single carrier of this variant, whereas the father was wild-type (**Figure 1C**). Bearing in mind the non-consanguinity of the parents, paternity tests to confirm family relationships were carried out. Given that CHS is an AR disorder, we conducted a SNP-array (Cytoscan HD, Affymetrix) according to manufacturer’s protocol (Affymetrix Santa Clara, C.A., U.S.A.). The chromosomal region containing the *LYST* gene was assessed for copy number variations (CNVs) and CN-LOH using Chromosome Analysis Suite software (Affymetrix) for both the patient and her parents. Filters applied for the detection of CNV were ≥ 20 consecutive markers in a region of at least 50kb, and for regions of CN-LOH, ≥ 100 markers in at least 5,000kb. Whilst no CNVs were observed in the parents’ DNA, a CN-LOH on the telomeric region of the long arm of chromosome 1 encompassing 25Mb (1q41q44) was detected in the child, accounting for the candidate variant in homozygosis in the *LYST* gene (**Figure 2**). A time-line summary of genetic investigations is represented in **Supplementary Figure 2**.

In addition, UPiD can alter the dosage of imprinted genes contributing to the phenotype of the disease (5, 17). Therefore, although the truncated LYST protein derived from the c.8380dupT variant would explain the severe phenotype in our patient, the possibility of an altered imprinted gene dosage as a consequence of the partial UPiD was also examined. We consulted the “Geneimprint” portal (http://www.geneimprint.com/site/genes-by-species) and found that *OBSCN* and *OR11L1* genes, in the region affected by the partial-UPiD, were predicted as paternally imprinted. However, the alteration of such genes cannot be related to the CHS patient phenotype.

The patient experienced a spontaneous recovery from the cytopenias. However, she was repeatedly admitted to the hospital due to frequent episodes of high fever, general condition impairment, and splenomegaly varying between 5 and 15 cm. Recurrence of exacerbated cytopenias required a transfusion of red blood cells in one occasion and G-CSF in two episodes. Although the patient did not meet the criteria for massive hemophagocytic lymphohistiocytosis (HLH) established by the 2004 Hystiocyte Society guidelines the last time she was hospitalized (**Table 1**), given the progression of the condition and clinical deterioration, she was treated with Dexamethasone and Etoposide according to the HLH protocol - 94/2004 (18, 19) to which she showed good response. Once remission was achieved, the patient received a stem cell transplant (HCT) from an unrelated HLA 10/10 compatible donor at 5^1/2^ years after following a conditioning regimen with busulfan, fludarabine and ATG. After 2 years since the transplant, the patient maintains complete donor chimerism, showing a normal physical and neurological development, and has not required subsequent admissions into the hospital.

**Table 1:** Patient’s clinical and treatment progression from the first admission to date.

**Reason for admission**	**Fever. Oral antibiotic rejection.**	**Acute tonsillitis.Vomiting.**	**EBV infection.Peritonsillar phlegmon.**	**High fever 72h**	**High fever 48h. Vomiting.**	**Fever 48 h.HSM increase.Food rejection.**
**Clinical laboratory**						
Leucocytes (cell/mm3)	2,930	1,830	6,460	3,740	3,070	7,030
Neutrophils (cell/mm3)	0	530	710	480	390	2,150
Hb (gr/dl)	9.7	9.7	8.4	9.7	6.6	10.6
Platelets (cell/mm3)	82,000	130,000	149,000	69,000	65,000	170,000
Fibrinogen (mg/dl)	NA	391	355	407	413	227
Ferritin (ng/mL)	NA	413	271	316	283	601
GOT (U/L)	59	39	35	218	22	NA
GPT (U/L)	43	NA	19	226	19	NA
LDH (U/L)	815	414	333	670	410	363
Triglycerides (mg/dl)	NA	68	259	304	246	NA
**Treatment**	Amox-clav	Amox-clav	Cefotaxime + Clindamycin Prednisone Acyclovir IVIg	Cefotaxime G-CSF IVIg	Cefotaxime + Amikacin IVIg G-CSF Dexamethasone	Dexamethasone Etoposide* IVIg
**Others**	Positive PCR for adenovirus ad RSV		EBV load: 2141 copies/mL		Blood culture: *Staphylococci hominis*	
**Age**	2 years 3 months	3 years 11 months	4 years 8 months	5 years 1 months	5 years 2 months	5 years 3 months

NA, Not available; Amox-clav, amoxicillin and clavulanate potassium; PCR, Polymerase Chain Reaction; RSV, Respiratory Syncytial Virus; IVIg, intravenous immunoglobulins; G-CSF, Granulocyte stimulating factor; EBV, Epstein-Barr virus. *First dosage at first admission. Five subsequent administered at the ambulatory.

## Discussion

This study reports the first case of CHS as a result of partial-UPiD. The patient harbored a novel pathogenic frameshift variant (NM_000081.3:c.8380dupT) in homozygosis in the *LYST* gene. To date, 72 mutations in this gene have been associated with CHS (20), and UPD has been reported as the causative mechanism only in three CHS cases (including the patient described here) (17, 21). A comparison of the three cases of UPD in CHS is shown in **Table 2**. In addition, 11 cases of primary immunodeficiencies caused by UPD have been reported to date (9) (considering this study) but only SCID (22) and LCK (23) in chromosome 1. Taken together, these data highlight the rarity of this event.

**Table 2:** Summary of reported cases of UPD in CHS with clinical and diagnostic tools detailed.

			**Patient 1 (** 17 **)**	**Patient 2 (** 21 **)**	**Patient 3^1^**
**Gender**			Male	Male	Female
**Age at diagnosis**			5 months old	6 years old	2 years old
**Severity**			Severe	Severe	Severe
**Type of UPD**			paternal UPhD	maternal UPiD	maternal partial-UPiD
**Mutation (NM_000081.3)**		**Coding impact**	nonsense	nonsense	frameshift
		**HGVS coding**	c.11002G>T	c.2621_2622delTTinsAA	c.8380dupT
		**HGVS protein**	p.E3668*	p.F874*	p.Y2794Lfs*8
		**Domains affected**	WD40	PH, BEACH and WD40	PH, BEACH and WD40
		**Zygosity**	homozygous	homozygous	homozygous
		**Exon**	50	6	32
**Consanguinity family**			No	No	No
**Clinical features**			Onset: eczema due to MRSA infection.	At diagnosis: oculocutaneous albinism, accelerated phase	Onset: adenovirus and RSV respiratory infections. At diagnosis: oculocutaneous albinism, accelerated phase
			At diagnosis: oculocutaneous albinism, accelerated phase		
**Physical development**			Delay in the acquisition of motor skills	Normal	Normal
**Neurocognitive development**			Delay (Denver Developmental Screening Test II)	Normal	Normal
**Morphology**			Giant granulations in leukocytes ^a^	Giant granulations in leukocytes ^a^	Giant granulations in leukocytes. Intense vacuolization and purple inclusion ^b^
**Hemogram alterations**			NA	NA	Hemoglobin: 9.7 g/dL. Leucocytes: 2.93x10^3^/µl – 0% neutrophils, 65.9% lymphocytes, 26.6% monocytes, 7.5% basophils. Platelets: 82x10^3^/µl
**Blood biochemical alterations**			Hypothyroidism (TSH 11.3 mU/L, T4 9.9 mcg/dl, negative thyroid antibodies.	NA	High CRP (48.9 mg/L), AST (59 U/L), ALT (43 U/L) and LDH (815 U/L).
**Immunophenotype analysis**			NA	NA	Available^2^
**Cytotoxic capacity**			Absent NK and CTL function.	NA	Low NK and CTL function.
**Diagnostic tools**	**LOH examination**		CGH, whole genome SNP-array and q-PCR.	SNP markers (1q42–q43 region)	SNP-array
	**Molecular results**		DNA sequencing of *LYST* exons and flanking introns	cDNA amplification, sequencing of abnormal transcript and PTT analysis	cDNA amplification and sequencing

^1^ Patient reported in this study.

^2^ See **Supplementary Table 1**.

a Examined in PB.

b Examined in PB and BM.

UPD, uniparental disomy; UPhD, uniparental heterodisomy; UPiD, uniparental isodisomy. HGVS, Human Genome Variation Society; MRSA, methicillin-resistant Staphylococcus aeureus; RSV, respiratory syncytial virus; NA, not available; TSH, thyroid-stimulating hormone; CRP, C-reactive protein; NK, natural killer; CTL, cytolytic T-lymphocyte; AST, aspartate transaminase; ALT, alanine aminotransferase; LDH, lactate dehydrogenase; LOH, loss of heterozygosity; CGH, comparative genomic hybridization; SNP, single nucleotide polymorphism; PTT, protein truncation test.

To assess the pathogenicity of the c.8380dupT variant and its association to the clinical phenotype of the patient, an *in silico* analysis was performed, suggesting that the LYST protein might be affected. The frameshift produced by the c.8380dupT leads to a PTC in exon 32 resulting in the loss of PH, BEACH and WD40 highly conserved domains at the C-terminal end of the protein. Both WD40 and PH domains are involved in targeting proteins to appropriate subcellular compartments; PH domain also participates in the protein-protein interaction with a binding partner. Therefore, the LYST protein would be truncated and non-functional (13). PTC could also initiate an mRNA degradation by the nonsense mediated decay (NMD), preventing its translation into the protein (24). However, the patient’s *LYST* RNA was successfully retro-transcribed into cDNA for molecular studies, suggesting that this transcript would escape the NMD surveillance mechanism. Additionally, we also amplified and sequenced the mother’s *LYST* cDNA region including the c.8380dupT mutation. The similar intensity of both alleles in the mother’s Sanger sequencing electropherogram rules out a significant degradation of the mutated allele (**Supplementary Figure 1B**). Therefore, all these data point out that the *LYST* c.8380dupT variant is pathogenic and would explain the severe CHS phenotype and childhood onset observed in our patient. In addition, although the genotype-phenotype correlation in CHS cases remains controversial, several studies have reported patients harboring frameshift, nonsense and splice-site mutations resulting in an absent LYST protein and similar clinical outcomes to that of our patient (10, 11). In contrast, the milder adult form of CHS is often due to missense mutation encoding for a partially functioning protein. However, Sánchez-Guiu et al. (25) demonstrated different outcomes due to a direct consequence of changes in protein structure and, especially, in the case of missense mutations, in electrostatic surface potential (25). Therefore, identifying the real molecular effect of the mutation is crucial to solve genotype-phenotype relationship.

Nevertheless, the detection of the c.8380dupT variant in homozygosity, the non-consanguinity of the parents and the results of segregation studies, showing the mother as the only heterozygous carrier, led us to consider the presence of other underlying genetic events, such as chromosomal deletion or loss of heterozygosity. Consequently, to determine the causative molecular mechanism of the disease, a SNP-array was performed. This analysis revealed that the patient harbored a CN-LOH affecting the 1q42.12 – 1q44 region, which suggests a partial-UPiD of maternal origin. In CHS, UPiD owing to CN-LOH has been previously identified (21), however, to our knowledge, this is the first CHS case reported as a consequence of partial-UPiD (17, 21). The two previously reported CHS patients have a severe phenotype at early stages caused by nonsense mutations. Manoli et al. (17) reported a paternal UPhD proband with additional developmental delays despite carrying the most distal mutation among the three cases (**Table 2**). The authors argued that this could be the result of additional non-detectable effects linked to a deregulation in genomic imprinting of chromosome 1 as previously suggested (26). Nonetheless, several AR disorders, including the other CHS case of maternal UPiD, have been purely described as a consequence of UPD of chromosome 1 without any atypical phenotype, suggesting the lack of imprinted genes on either the paternal or maternal alleles (21, 27). In our case, although partial-UPiD encompassed 93 OMIM genes besides *LYST*, of which *OBSCN* and *OR11L1* (http://www.geneimprint.com/site/genes-by-species) were predicted to be paternal imprinted genes, only *OBSCN* has been related to an AR disorder (i.e. fibromuscular dysplasia and Limb-Girdle muscular dystrophy) (28). Since our patient exhibited no other uncommon conditions other than classic CHS, we concluded that the alteration of such genes is not related to the patient phenotype.

Although there is no clear explanation for the mechanism that leads to partial-UPiD observed in our patient, this could be produced by an early mitotic division error resulting in the loss of the paternal chromosome 1 segment, as indicated in previous reports (3, 29). In other AR diseases (30–32), partial-UPiD has been associated with mosaicism as a very rare event (33). However, the SNP-arrays technology analyses may have disregarded the presence of mosaicism in our patient, especially if we consider that the sensitivity of this technique is 10%, as well as the premature alteration in mitotic division (34).

On the other hand, CHS is also associated with the development of HLH, which affects more than 75% of CHS patients within their first decade and it is fatal unless treated (35). HLH is a main characteristic of the ‘accelerated phase’ of the disease, hence, being particularly useful when determining the need for a HCT. A decrease in NK-cell and cytotoxic T lymphocytes capacity provides a diagnostic criteria and predictor of the severity and transformation to HLH (36). In addition to function impairment in NK cells, our patient displayed a clinical deterioration with consecutives hospitalization matching HLH-94/2004 guidelines. Therefore, since transplantation appears to be most successful if performed prior to the onset of HLH or during remission, we decided to perform HCT once remission was achieved. Although a HCT does not prevent the possible neurologic detriment, our patient remains healthy after two years from the HCT, showing a normal immunologic, hematologic and neurological development.

In summary, we report the first CHS case with a pathogenic variant in homozygosis as a consequence of partial-UPiD. Risk of AR conditions, such as CHS, are easily estimated with siblings having a 25% risk of developing the disease. However, the prevalence of germline UPD is harder to quantify with frequencies from 0.03 – 0.3% (37–40), being partial-UPiD significantly lower (40). Despite the low risk of recurrence, having detected the heterozygosis c.8380dupT variant in the mother of this child and defined the inheritance mechanism, will allow a more accurate genetic counseling for future pregnancies and other family members at risk. Taken together, in non-consanguineous families, UPD should be considered as a potential mechanism of CHS when a homozygous mutation is identified. This case also underlines the pivotal role of SNP-array as a diagnostic approach to assess the molecular mechanism responsible of AR disorders, such as CHS.

## Data Availability Statement

The raw data supporting the conclusions of this article will be made available by the authors, without undue reservation.

## Ethics Statement

The studies involving human participants were reviewed and approved by Comité de Ética de la Investigación con medicamentos del Hospital Universitario y Politécnico la Fe. Written informed consent to participate in this study was provided by the participants’ legal guardian/next of kin.

## Author Contributions

MB-N analyzed molecular studies and wrote the paper. MI and AL supervised experimental findings. CF-J and MM-G performed and analyzed the functional assays research and data analysis. HR-V, JT and CC were involved in the management of the patient. ES performed cytogenetic analysis. ÁZ supervised clinical and experimental findings. RC performed and analyzed the molecular studies. JC and RC were responsible for the conception of the study and final approval of the draft. All authors contributed to the article and approved the submitted version.

## Funding

This study was supported by research funding from FEDER funds (CIBERONC, CB16/12/00284), Instituto de Salud Carlos III grants PI16/01113, PI17/00660, PI18/1472, PI19/00812 cofinanced by the European Regional Development Fund (ERDF); as well as from the “Conselleria de Educación, Cultura y Deporte” GV/2019/084. MB-N and AL are recipients of a fellowship from the “Asociación Española Contra El Cáncer” and the “Fundación Española de Hematología y Hemoterapia”, respectively.

## Conflict of Interest

The authors declare that the research was conducted in the absence of any commercial or financial relationships that could be construed as a potential conflict of interest.
