# Supplementary material for: Case Report: Partial Uniparental Disomy Unmasks a Novel Recessive Mutation in the LYST Gene in a Patient With a Severe Phenotype of Chédiak-Higashi Syndrome
Source: Front Immunol. 2021 Mar 31;12:625591. doi: 10.3389/fimmu.2021.625591 (PMC8044466; doi:10.3389/fimmu.2021.625591)
Supplement: Supplementary file 1 [file Presentation_1.pdf]

Supplementary Figures and Tables

A B

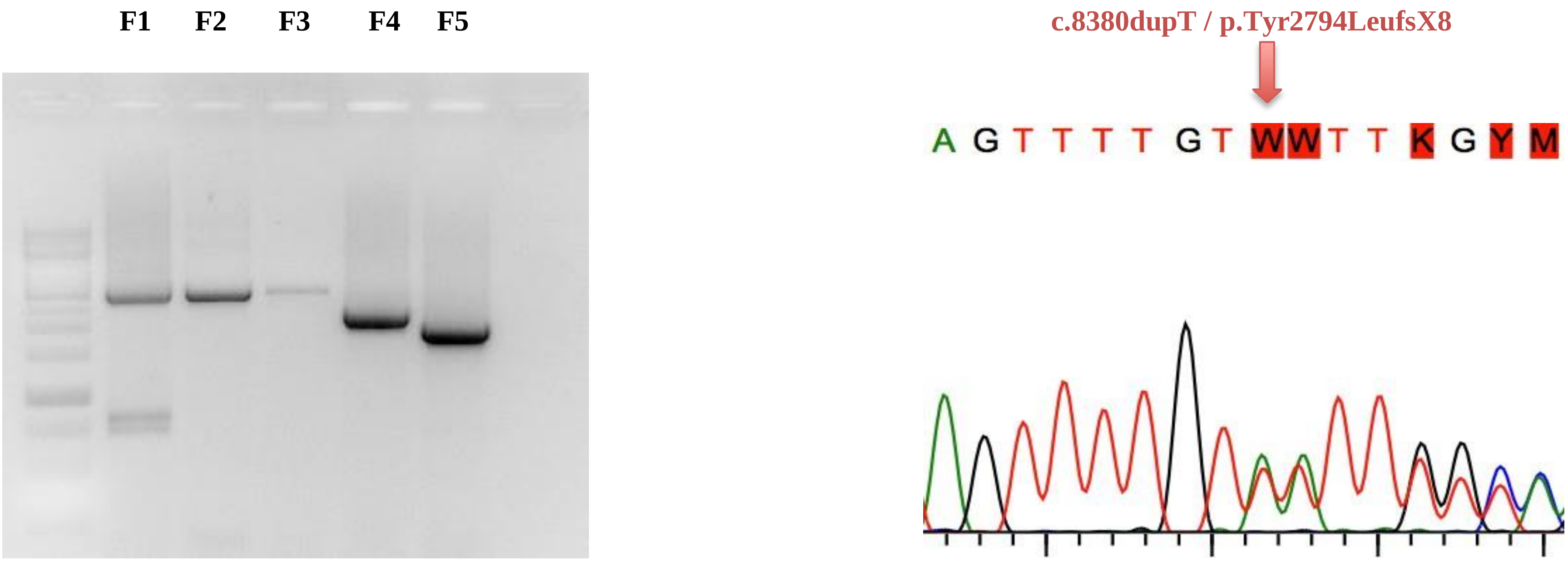

Patient: LYST cDNA amplification

Mother: LYST cDNA sequencing

Supplementary Figure 1. LYST cDNA amplification and sequencing. **A)** 0,8% agarose gel showing the *LYST* amplification from patient's cDNA. The bands for the 5 overlapping fragments correspond to the expected size: F1, 2915 bp; F2, 2938 bp; F3, 3064 bp; F4, 2170 bp; F5, 1853 bp. The different band intensity is due to different amplification efficiency and it is equivalent to a healthy control (data not shown). **B)** Sanger sequencing of the mother's cDNA showing the c.8380insT mutation (the mother is an heterozygous carrier). The correct amplification of patient's cDNA and the similar intensity of both alleles in the mother's Sanger sequencing electropherogram rule out a significant degradation of the mutated allele.

	Cytogenetics	Molecular Diagnosis – *LYST* interrogation			Segregation analysis and copy number evaluation		
**Genetic investigations**	Pseudo Chédiak Higashi-like inclusions	cDNA amplification and sequencing	gDNA confirmation	Databases quering and *in silico* protein assessment	Detection of *LYST* mutation in parents	Paternity tests	SNP-array
**Purpose**	Differential diagnosis	Chédiak-Higashi Syndrome diagnosis			Inheritance pattern		
**Findings**	Normal KT	*LYST* c.8380dupT (NM_000081.3) in homozygosis			Mother as the only carrier in heterozygosis<br>CN-LOH in the patient (1q41q44)		

Supplementary Figure 2. Time-line summary of genetic investigations and results obtained for each diagnosis stage. KT: karyotype; cDNA: complementary cDNA; gDNA: genomic DNA; CN-LOH: copy neutral loss of heterozygosity.

Supplementary Table 1. Distribution and phenotypic characteristics of different compartments of hematopoietic precursors BM cells in normal *versus* the patient's BM.

		Distribution of cell population (%)	
**BM cell subsets**	**Phenotype**	**Normal BM** (1,2)	**Patient's BM**
**Total count of lymphoid cells**	NA	8.6 - 23.8%	8.5%
**% Total BM CD34^{+} precursors**	NA	0.9% (0.2–1.6%)	2.5%
**% BM CD34^{+} B precursors**	nuTdT+ cyCD79a+ CD19+	23% (<1-45%)	45%
**% BM neutrophil lineage** (myelocytes)	CD34^{-}/CD13^{+}/CD11b^{-}	11% (3-25%)	26%
**Monocytic precursors** (monoblasts)	CD34^{-}/CD64$^{+/hi}$/CD14^{-}	10% (5-16%)	1.8%
**Monocytic precursors** (promonocytes)	CD34^{-}/CD64^{hi}/CD14^{+}	4% (2-6%)	4.2%

Results expressed as mean and range between brackets. NA: not applicable; BM: bone marrow.

Supplementary Table 2. Primers and PCR conditions for cDNA *LYST* amplification.

Fragment	Primer name	Primer sequence	Amplimer length	Ta	Extension	Cycles
F1	LYST F1 forward	GCCACAAACCAGGTGAAGCTTT	2915 bp	66 °C	3 min	45
	LYST F1 reverse	GCTCGCTGGCTGTGCTGTCATA				
F2	LYST F2 forward	ATGCCTCAGTTCCAGATATTGA	2938 bp	66 °C		
	LYST F2 reverse	CCAGTTCCACCAATTTCGT				
F3	LYST F3 forward	GGCAAGCCAGTCAATGACTAC	3064 bp	66 °C		
	LYST F3 reverse	TGGCTTTACTTGAACCAATAGA				
F4	LYST F4 forward	CAAGCAACTGAAACGGAACTT	2170 bp	66 °C		
	LYST F4 reverse	GACAGATGTTCTGCGACACGTAG				
F5	LYST F5 forward	CGTGCAGCCCTATCACTA	1853 bp	59 °C		
	LYST F5 reverse	ACTTTATCATTATTTGGATGGTT				

Ta: Annealing temperature; bp: base pairs.

Bibliography – Supplementary Material

1. Orfao A, Matarraz S, Pérez-Andrés M, Almeida J, Teodosio C, Berkowska MA, van Dongen JJM. Immunophenotypic dissection of normal hematopoiesis. *J Immunol Methods* (2019) **475**:112684. doi:10.1016/j.jim.2019.112684

2. Matarraz S, López A, Barrena S, Fernandez C, Jensen E, Flores-Montero J, Rasillo A, Sayagues JM, Sánchez ML, Bárcena P, et al. Bone marrow cells from myelodysplastic syndromes show altered immunophenotypic profiles that may contribute to the diagnosis and prognostic stratification of the disease: A pilot study on A series of 56 patients. *Cytom Part B - Clin Cytom* (2010) **78**:154–168. doi:10.1002/cyto.b.20513
